# Estimation of Two Diuretics Using Fluorescent Nitrogen Doped Carbon Quantum Dots: Application to Spiked Human Plasma and Tablets

**DOI:** 10.1007/s10895-023-03217-z

**Published:** 2023-03-31

**Authors:** Mona H. Abo Zaid, Nahed El-Enany, Aziza E. Mostafa, Ghada M. Hadad, Fathalla Belal

**Affiliations:** 1 Pharmaceutical Chemistry Department, Faculty of Pharmacy, Delta University for Science and Technology, Gamasa, 35712 Egypt; 2 Pharmaceutical Analytical Chemistry Department, Faculty of Pharmacy, Suez Canal University, Ismailia, 41522 Egypt; 3 Pharmaceutical Analytical Chemistry Department, Faculty of Pharmacy, Mansoura University, Mansoura, 35516 Egypt; 4 Pharmaceutical Chemistry Department, Faculty of Pharmacy, New Mansoura University, New Mansoura, 7723730 Egypt

**Keywords:** Sucrose, Urea, Quantum dots, Eplerenone, Spironolactone, Spiked human plasma

## Abstract

Highly fluorescent nitrogen doped carbon quantum dots (N-CQDs) were prepared by a single-step method based on microwave heating of cane sugar and urea. The produced N-CQDs were applied as nano-sensors for the spectrofluorimetric determination of eplerenone and spironolactone. A strong emission band at 376 nm was obtained after excitation at 216 nm due to the produced N-CQDs. The native fluorescence of N-CQDs was obviously quenched upon adding increased concentrations of each drug. A strong correlation was found between the fluorescence quenching of N-CQDs and the concentration of each drug. The method was found to be linear over the range of 0.5 to 5.0 μg/mL for eplerenone and 0.5 to 6.0 μg/mL for spironolactone with LOQ of 0.383 μg/mL and 0.262 μg/mL. The developed method was further extended for determination of both drugs in their pharmaceutical tablets and spiked human plasma. The results obtained were statistically compared with those of reported methods. The mechanism of fluorescence quenching of N-CQDs by the two drugs was discussed.

**Supplementary Information:**

The online version contains supplementary material available at 10.1007/s10895-023-03217-z.

## Introduction

Eplerenone (EPL) and spironolactone (SPR) belong to the class of potassium-sparing diuretics. Both drugs are prescribed for hypertension and heart failure. Eplerenone chemically is: 9,11α-Epoxy-7α-(methoxycarbonyl)-3-oxo-17α-pregn-4-ene-21,17-carbolactone [1] (Supplementary materials Fig. S1). It is an aldosterone receptor antagonist which helps in reducing the risk of myocardial infarctions and lowering blood pressure [2]. Spironolactone chemically is: (2`R)-7α-(Acetylsulfanyl)-3`,4`-dihydro-5`H-spiro[androst-4-ene-17,2`-furan]-3,5`-dione (Supplementary materials Fig. S2) [1]. It is necessary to observe their plasma levels mainly in patients who suffer from kidney problems as both drugs can cause serious complications. Many analytical methods for EPL estimation were reported, including spectrophotometry [3–5], LC–MS [6], RP-HPLC [7–9], HPTLC [10] and spectrofluorimetry [11]. Many analytical techniques have been used for the estimation of SPR, either in pharmaceutical preparations or biological fluids included; spectrophotometry [12–14], HPLC [15–17], HPTLC [18] and spectrofluorimetry [19]. Most of these analytical methods are multi-steps procedures and require expensive instruments (Table 1). Nowadays, nano-sensors and their pharmaceutical applications gain the greatest interest. For many years, semiconductor quantum dots have been extensively investigated for their strong and tunable fluorescence emission properties, which enable their applications in biosensing and bioimaging. However, semiconductor quantum dots possess certain limitations such as high toxicity due to the use of heavy metals in their production. It is known that heavy metals are highly toxic even at relatively low levels, which may prove prohibitive to any clinical studies. This prompted the creation of CQDs to replace semiconductor quantum dots [20]. Carbon quantum dots (CQDs) are classified as fluorescent nano particles which are characterized by their low cost, photostability, biocompatibility, high water solubility, safety and being nontoxic [21–24]. Moreover, doping of heteroatoms as nitrogen atom to the structure of CQDs enhances the optical and electrical properties of CQDs [25, 26].

**Table 1:** Comparison between the proposed method and reported methods

	Matrix	Analytical Technique	Detection	Linear Range	Ref
EPL	Raw material, tablets and spiked human plasma	Spectrofluorimetric method	Fluorescence Detection by measuring emission at 376 nm after excitation at 216 nm	0.5–5 µg/mL	This method
	Raw material and tablets	Spectrophotometric method	UV at 245 nm	5-15 µg/ml	3
	Raw material and tablets	Spectrophotometric method	Derivatization with 2, 4-dinitrophenyl hydrazine then measuring absorbance at 430 nm	5- 35 µg /ml	4
	Raw material and tablets	Spectrophotometric method	Measuring absorbance at 241 nm	4—24 µg/mL	5
	Human urine	LC/MS	m/z 415 → 163	50–10,000 ng/ml	6
	Raw material and commercial tablets	RP-HPLC	UV Detection at 242 nm	0.1–40 µg/mL	7
	Spiked human plasma	RP-HPLC	UV Detection at 241 nm	100–3200 ng/mL	8
	Raw material and tablets	RP-HPLC	UV Detection at 240 nm	10–100 μg/mL	9
	Raw material and tablets	HPTLC	Plates scanned at 242 nm	5–600 ng/band	10
	Raw material, tablets and spiked human plasma	Spectrofluorimetric method	Fluorescence detection by measuring emission at 446 nm after excitation at 370 nm	0.2–3.0 μM	11
SPR	Raw material, tablets and spiked human plasma	Spectrofluorimetric method	Fluorescence Detection by measuring emission at 376 nm after excitation at 216 nm	0.5–6 µg/mL	This method
	Raw material and tablets	Spectrophotometric method	UV detection at 237	5–35 μg/ml	12
	Raw material and commercial tablets	Spectrophotometric method	Isosebtic point and ratio subtraction	3–50 µg/mL	13
	Pharmaceutical Formulation	Spectrophotometric method	Derivative ratio spectra at 237 nm	5–40 µg/mL	14
	Raw material and pharmaceutical preparation	RP-HPLC	UV detection at 230 nm	5.0–50 μg/mL	15
		TLC	Plates scanned at 235 nm	0.3–1.5 µg/band	
	Raw material and tablets	RP-HPLC	UV detection at 236 nm	25–150 µg/mL	16
	Raw material, tablets and human urine	RP-HPLC	UV detection at 235 nm	0.5–10.0 μg/mL	17
	Raw material, tablets and spiked human plasma	RP-HPLC	UV detection at 254 nm	2–60 μg/mL	18
		HPTLC	Plates scanned at 254 nm	0.05–2.6 µg/band	
	Raw material and tablets	Spectrofluorimetric method	Fluorescence detection at 587 nm after excitation at 388 nm	2.5–700 µg/mL	19

The current work aimed to develop a sensitive spectrofluorimetric method for determination of EPL and SPR in commercial tablets and spiked human plasma with no need for any derivatization. The method of preparation of N-CQDs depends on microwave heating of aqueous solution of cane sugar as carbon source and urea as nitrogen source. Both drugs showed quantitative quenching of the fluorescence of N-CQDs.

The main advantage of the proposed method is the ability of the method to produce N-CQDs with quantum yield of 0.57 in less than thirty minutes and its ability to detect very low concentrations of the studied drugs.

## Experimental

### Reagents and Materials

- Methanol (HPLC grade) was obtained from Fisher in Belgium.
- Glacial acetic acid (99%) was obtained from Alfa chemical in Egypt.
- Boric acid and orthophosphoric acid (85%) were obtained from Sigma-Aldrich in Switzerland.
- Britton Robinson Buffer was made up of mixture of 0.04 M boric acid, 0.04 M acetic acid and 0.04 M phosphoric acid, then 0.2 M sodium hydroxide was used to adjust pH range from 2.1 to 12.
- Deionized water was used during the course of the work.
- Spironolactone (purity of 99.9%) was obtained from Kahira Pharm. &Chem. Company Cairo in Egypt.
- Eplerenone (purity of 99.9%) was kindly provided by NODCAR, Cairo, Egypt.
- From local pharmacies; Tensopleron 25 mg tablets (B.N.125304) and Tensopleron 50 mg tablets (B.N.125402), products of Global Napi Pharmaceuticals, Spectone® 25(Batch N0.2110863) and Spectone® 100 mg (Batch N0.2110871) products of Kahira Pharm. & Chem. Company, Cairo, Egypt.
- Human plasma samples were provided by Mansoura University Hospital, Mansoura, Egypt. The samples kept frozen until use at − 80 °C.

### Apparatus

- Shimadzu RF-6000 series Spectrofluorometer equipped with Xenon lamp (150 W) has been used for fluorescence measurements in the wavelength range from 200 to 600 nm. The apparatus was operated at high sensitivity mode during the entire work.
- JEM-2100 high-resolution transmission electron microscope (HRTEM) (JEOL, Tokyo) operated at 200 kV to investigate the morphology of N-CQDs.
- FT-IR Spectrometer (Thermo Scientific Nicolet—iS10) (MA, USA).
- Shimadzu UV-1900i Spectrophotometer (Japan) has been used for spectrophotometric measurements in the wavelength range from 200 to 600 nm.
- Elmasonic S100 (H) Ultrasonic bath (Germany).
- ZX3 vortex mixer (Velp. Scientific) (Italy).
- Jenway 3510 pH-Meter (England).
- Centrifuge, model 2-16P (Germany).
- Domestic LG microwave (Frequency of 2,450 MHz and output of 900 W) (Model No: MH7043BARS) has been used for synthesis of N-CQDs.

### Preparing Solutions

Preparing stock solutions (1000.0 µg/mL) of EPL and SPR was carried out by dissolving 10.0 mg of each drug separately in 10.0 mL of methanol. Stock solutions were diluted by one-tenth to prepare working solutions (100.0 µg/mL). The working solutions were then used to prepare different concentrations.

### Synthesis of fluorescent N-CQDs

A domestic microwave was used to prepare the N-CQDs by heating fifteen grams of cane sugar and three grams of urea dissolved in 30.0 mL deionized water. The solution was heated for ten minutes till complete charring. Then, the formed N-CQDs were left at room temperature to cool down. Water was used to dilute the produced N-CQDs. Centrifugation was carried out at 6000 rpm for ten minutes to remove suspended particles. Then, the impurities were removed by 0.22 µm Syringe filters. The N-CQDs were transferred to 100.0 mL volumetric flask and the volume was completed to the mark with deionized water to prepare stock solution.

### Quantum Yield

The single point method was used to calculate the quantum yield of N-CQDs according to Eq. 1 [27]:

1 $${\Phi }_{\mathrm{x}}={\Phi }_{\mathrm{st}}\times \left({\mathrm{F}}_{\mathrm{x}}/{\mathrm{F}}_{\mathrm{st}}\right)\times \left({\upeta }_{\mathrm{x}}/{\upeta }_{\mathrm{st}}\right)\times \left({\mathrm{A}}_{\mathrm{st}}/{\mathrm{A}}_{\mathrm{x}}\right)$$

In which:

Φ represents the quantum yield, F, A and η stand for the integrated measured intensity of emission, the absorbance and the solvent refractive index. The subscripts x refers to the unknown sample and st refers to the reference samples. In aqueous solutions, η_x_/η_st_ is equal to 1. Quinine sulfate was used as the standard and dissolved in 0.1 M H_2_SO_4_ (QY: 0.54). The produced N-CQDs exhibit high quantum yield (0.57).

### General Procedures

#### Raw Materials

In this study, different aliquots from working standard solutions (100.0 μg/mL) of both drugs were transferred to a series of 10.0 mL volumetric flasks containing 400 μL aliquots of N-CQDs. 1 mL of BRB of pH 3.0 for EPL and 1 mL of BRB of pH 5.0 for SPR were added to each flask. Deionized water was used to complete each solution to the mark. To create the calibration curves, the quenching values of fluorescence (ΔF) at 376 nm were plotted against final drug concentrations in (μg/mL). As an alternative, the regression equations were derived.

#### Commercial Tablets

##### Tensopleron Tablets

Ten Tensopleron tablets were weighed and pulverized. An amount equivalent to 10.0 mg of EPL was transferred into a 10.0 mL volumetric flask then five milliliters methanol were added. Sonication was performed for thirty minutes. The content of the flask was completed to the volume with methanol and filtered to give a solution of 1000.0 μg/mL. Then 1.0 mL was transferred from the stock solution to 10.0 mL volumetric flask and diluted with methanol to give solution of 100.0 μg/mL. The procedure described above (“Raw Materials” section) was applied. Percentage recoveries were determined using the corresponding regression equation.

##### Spectone® Tablets

Ten Spectone® tablets were weighed and pulverized. An amount equivalent to 10.0 mg of SPR was accurately transferred into 10.0 mL volumetric flask then five milliliters of methanol were added. Sonication was performed for thirty minutes. The content of the flask was completed to the volume with methanol and filtered to give a solution of 1000.0 μg/mL. Then 1.0 mL was transferred from the stock solution to 10.0 mL volumetric flask and diluted with methanol to give solution of 100.0 μg/mL. The procedure was completed as described under “Raw Materials” section. Percentage recoveries were determined using the corresponding regression equation.

#### Spiked Human Plasma

Aliquots of EPL and SPR standard working solutions were accurately added to 1.0 mL human plasma in a series of 15.0 mL centrifuge tubes. After vortexing each tube for thirty seconds, the volume was made up to 5.0 mL with acetonitrile. Then centrifugation was performed at 3600 rpm for thirty minutes. Different volumes of the supernatant were transferred into 10.0 mL volumetric flasks and the described procedure (“Raw Materials” section) was followed. The percentage recoveries were calculated adopting the corresponding regression equation.

## Results and Discussion

The recent study presents rapid, simple, economic and sensitive spectrofluorimetric method for quantitative estimation of both EPL and SPR. The method of synthesis of N-CQDs is ecofriendly, rapid, facile and need simple procedure and available precursors. The method of preparation needs thirty minutes to yield hydrophilic and highly fluorescent probe which could be directly used in estimation of EPL and SPR.

### Characterization of N-CQDs

**Scheme 1:**
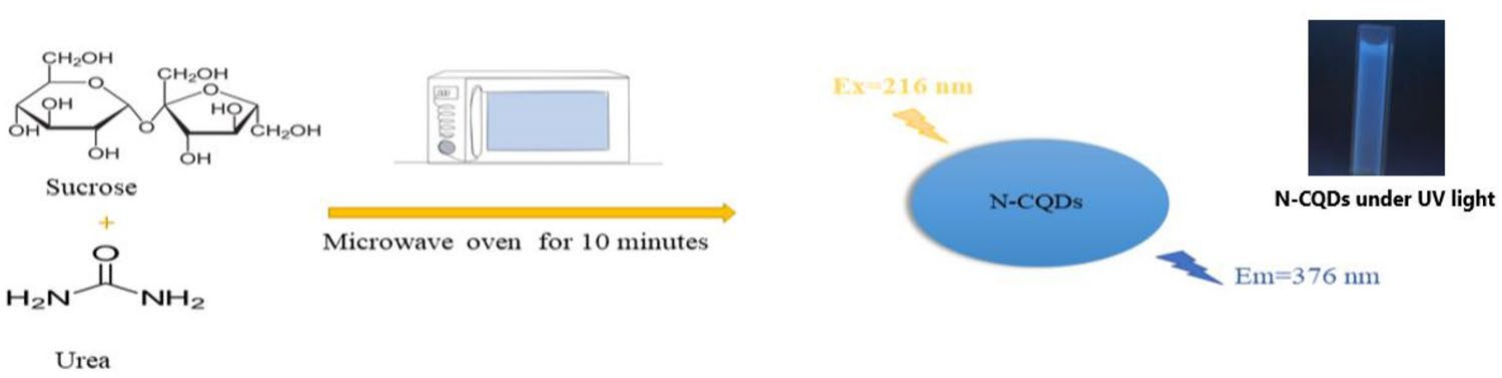
General procedure for synthesis of N-CQDs

**Fig. 1:**
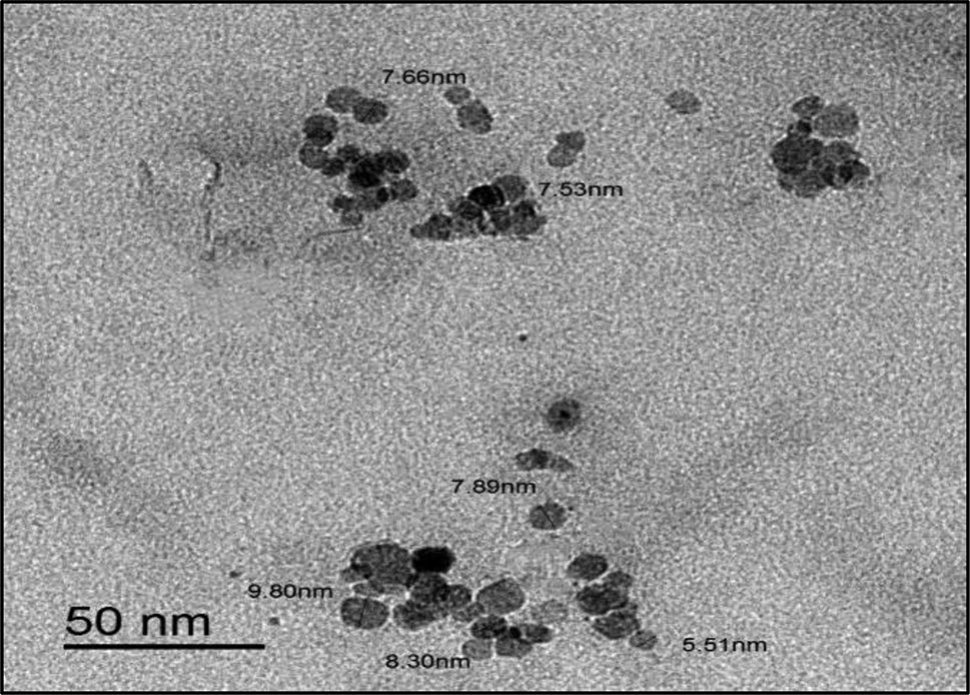
The typical HRTEM images of the N-CQDs

**Fig. 2:**
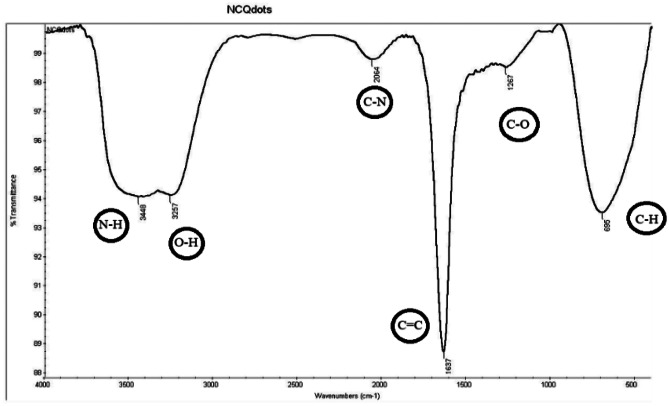
FT-IR spectra of N-CQDs

**Fig. 3:**
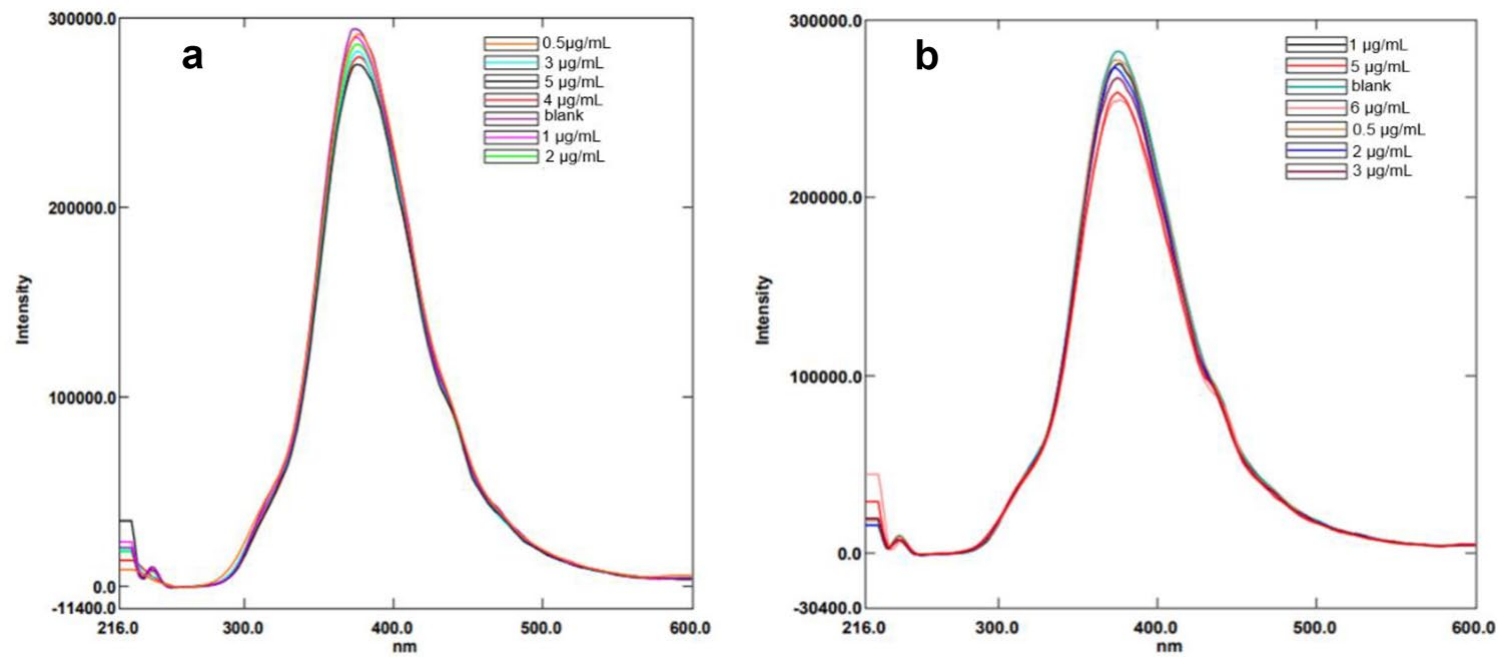
**a** Fluorescence emission spectra of the N-CQDs in aqueous solution upon addition of various concentrations of EPL (from top to bottom: 0 μg/mL, 0.5 μg/mL, 1.0 μg/mL, 2.0 μg/mL, 3.0 μg/mL, 4.0 μg/mL, 5.0 μg/mL), **b**. SPR (from top to bottom: 0 μg/mL, 0.5 μg/mL, 1.0 μg/mL, 2.0 μg/mL, 3.0 μg/mL, 5.0 μg/mL, 6.0 μg/mL)

**Scheme 2:**
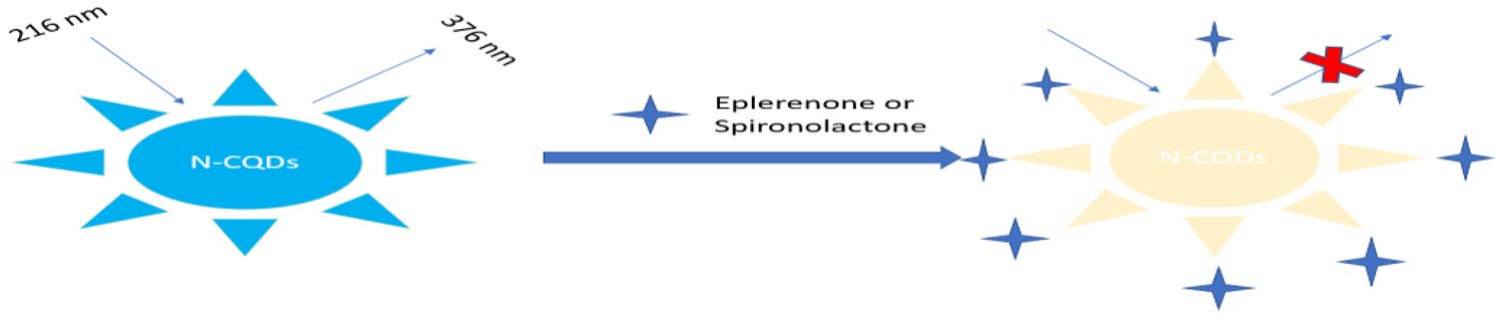
Scheme showing fluorescence quenching of N-CQDs by EPL and SPR

Microwave assisted method was used for synthesis of water-soluble N-CQDs using cane sugar and urea as carbon and nitrogen sources. A solution of orange color was produced indicating the formation of N-CQDs. The general procedure for synthesis of N-CQDs is illustrated in (Scheme 1). The N-CQDs showed spherical particles with diameters in the range of 5.5 nm to 9.8 nm with an average diameter of 7.7 nm as presented in TEM image (Fig. 1). FT-IR analysis was also used to identify functional groups of N-CQDs (Fig. 2). The broad band in the range of 3600– 3100 cm^−1^, the peaks at 2064, 1638 cm^−1^ and the vibrations at 1265 cm^−1^ represent N–H and O–H groups, C = C/C = O, C-N and the stretching modes of C–O–C band, respectively [28]. Also, the peak at 695 cm^−1^ represents C-H. The absorption spectrum of N-CQDs is shown in (Supplementary material Fig. S3). The excitation and emission spectra of the fluorescent N-CQDs are presented in (Supplementary Material Figs. S4 and S5), high fluorescence intensity of N-CQDs at λem = 376 nm (λex = 216 nm) and the quenching effect of each of EPL and SPR on N-CQDs are obvious as shown in (Fig. 3a and b). The fluorescence quenching of N-CQDs by EPL and SPR is illustrated in (Scheme 2).

### Mechanism of fluorescence response of N-CQDs to both EPL and SPR

Stern–Volmer’s equation was applied to analyze the fluorescence emission intensity of the N-CQDs-EPL and N-CQDs-SPR systems for a better understanding of the fluorescence quenching mechanism [29].

2 $${\mathrm{F}}^{0}/\mathrm{F}=1+{\mathrm{K}}_{\mathrm{q}}{\uptau }^{0}\left[\mathrm{Q}\right]=1+{\mathrm{K}}_{\mathrm{sv}}\left[\mathrm{Q}\right]$$

In this equation, F and F^0^ refer to the fluorescence intensities in the presence and absence of quencher, respectively. K_q_ and K_sv_ stand for the quenching rate and the Stern–Volmer quenching constants, τ^0^ is the average fluorescence lifetime (10^− 8^ s) and [Q] is the concentration of the quencher.

The quenching experiments were performed at 298 K. The quenching results were applied to the previous equation resulted in K_q_ values of 5.7 × 10^11^ for EPL and 1.06 × 10^12^ for SPR. The two values were larger than (2.0 × 10^10^ L.mol^−1^.s^−1^) which is the maximum diffusion rate constant [30]. As a result, the quenching mechanism is assumed to be static.

In this study, an overlap exists between the excitation spectrum of NCQDs and UV–VIS absorption spectrum of EPL and SPR (Supplementary material Fig. S6, S3). Therefore, Inner Filter Effect (IFE) might occur. The correction of N-CQDs fluorescence intensity for potential IFE was studied. The following equation was used to calculate the corrected fluorescence intensity Eq. (3) [31]:

3 $${\mathrm{F}}_{\mathrm{corr}}={\mathrm{F}}_{\mathrm{obs}}{\mathrm{X}10}^{ \left({\mathrm{A}}_{\mathrm{ex}}+{\mathrm{A}}_{\mathrm{em}}\right)/2}$$

In which:

F_obs_ represents the observed fluorescence intensity, F_corr_ is the corrected fluorescence intensity after removing inner filter effect from F_obs_, A_em_ and A_ex_ are the absorbance of the drug at the emission and excitation wavelengths of N-CQDs. Equation 4 was used to calculate the suppressed efficiency (%E) for observed and corrected fluorescence intensities.

4 $$\mathrm{\%E}=\left[1-\left(\mathrm{F}/{\mathrm{F}}_{0}\right)\right]\times 100$$

**Fig. 4:**
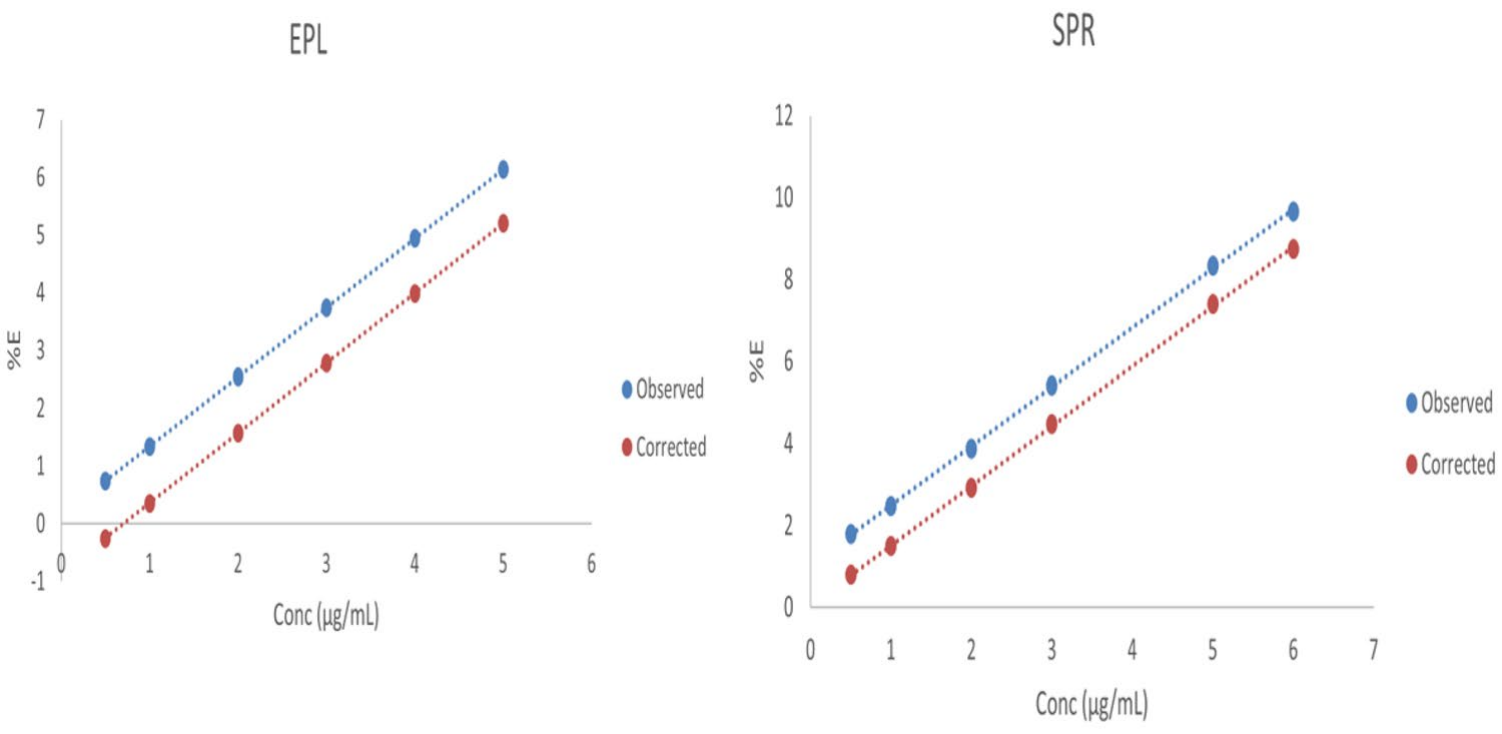
Plots of %E against concentrations of EPL and SPR

The plot between %E against drug concentration showed that IFE affect by 15.3% and 9.36% in the quenching of N-CQDs fluorescence intensity by EPL and SPR (Fig. 4).

### Performance Optimization

Different factors that affect the fluorescence sensing of EPL and SPR have been investigated to choose the optimum conditions.

#### Effect of pH and Volume of the Buffer

The influence of different volumes of BRB solution on fluorescence intensity quenching by EPL and SPR over pH range of 2.1–12 was studied. The highest ΔF value was achieved using 1 mL of the buffer of pH 3 for EPL and 1 mL of BRB of pH 5 for SPR (Supplementary material Fig. S7).

#### Effect of volume of N-CQDs

The maximum quenching of fluorescence intensity was attained by using a volume of 0.4 mL of N-CQDs with both drugs (Supplementary material Fig. S8).

#### Effect of Temperature and Incubation Time

The experiment was tried at different temperatures (298, 303 and 313 K) and the fluorescence intensities were recorded at the emission wavelength. It was noticed that there was no significant difference in the relative fluorescence intensities, so the experiment was carried out at 298 K (Supplementary material Fig. S9). Also, fluorescence spectra after adding the studied drugs to N-CQDs were recorded at different time intervals starting from one to forty minutes. It was found that the reaction between N-CQDs and the studied drugs was instantaneous and completed in nearly one minute. The fluorescence values were constant for thirty minutes.

### Method Validation

ICHQ_2_(R_1_) Guidelines were followed to prove that the method is valid [32].

The range and linearity of the suggested method were evaluated by plotting relative quenched fluorescence intensities versus concentrations of each drug in μg/mL. The method was found to be linear over the range of 0.5 to 5.0 μg/mL for EPL and 0.5 to 6.0 μg/mL for SPR (Supplementary material Fig. S10). The following equations represent the linear regression, Eqs. (5) and (6):

5 $$\begin{array}{ccc}\left({\mathrm{F}}_{0}-\mathrm{F}\right)/{\mathrm{F}}_{0}=0.012\mathrm{C}+0.0015& \left({\mathrm{R}}^{2}=0.9994\right)& \mathrm{for \ EPL}\end{array}$$

6 $$\begin{array}{ccc}\left({\mathrm{F}}_{0}-\mathrm{F}\right)/{\mathrm{F}}_{0}=0.014\mathrm{C}+0.011& \left({\mathrm{R}}^{2}=0.9998\right)& \mathrm{for \ SPR}\end{array}$$

where C is the concentration of each drug in μg/mL and F_0_, F are the fluorescence intensity of N-CQDs in the absence and presence of both drugs, respectively.

The proposed method is adequately sensitive for quantitative estimation of the analytes in biological fluids due to the relatively small values of LOD and LOQ (Table 2).

**Table 2:** Analytical performance data for the proposed method

	EPL	SPR
Linearity range (µg/mL)	0.5–5.0	0.5–6.0
Limit of detection (LOD)	0.126	0.087
Limit of quantitation (LOQ)	0.383	0.262
Regression equation	(F_0_-F)/F_0_ = 0.012C + 0.0015	(F_0_-F)/F_0_ = 0.014C + 0.011
Correlation coefficient	0.9997	0.9998
S.D. of residuals (Sy/x)	0.000593	0.000531
S.D. of intercept (Sa)	0.000461	0.000382
S.D.of slope (Sb)	0.000152	0.000108

The calculated mean % recoveries of raw materials of EPL and SPR within the specified concentration ranges for the comparison and proposed method guaranteed the accuracy of the method. The comparison method for EPL [5] depends on absorbance measurement at 241 nm in (30% v/v) methanol. While the comparative method for SPR [12] is based on measuring its absorbance in methanol at 237 nm. Variance F-test and Student’s t-test were applied to evaluate the results attained from the proposed method and the comparative method as presented in (Supplementary material Table S1) [33]. There was no significant difference between the methods in terms of precision and accuracy.

The intra-day and inter-day precisions were investigated to ensure the precision of the method. The values of %RSD were calculated using three different concentrations and each concentration was replicated three times. Both drugs showed values of % RSD less than 2.0% (Table 3).

**Table 3:** Intraday and inter-day precision data for the determination of the studied drugs by the proposed method

Analyte	Conc. taken in µg/mL	Intraday ^a^			Inter-day ^b^		
		Conc found ± S.D. (µg/mL)	%RSD	%Error ^c^	Conc found ± S.D. (µg/mL)	%RSD	%Error ^c^
EPL	1.0	1.0 ± 0.28	0.28	0.16	1.0 ± 0.88	0.88	0.51
	2.0	2.0 ± 0.71	0.71	0.41	2.0 ± 0.77	0.77	0.44
	3.0	3.0 ± 0.65	0.64	0.37	3.0 ± 0.83	0.83	0.48
SPR	1.0	1.0 ± 0.67	0.67	0.39	1.0 ± 0.91	0.91	0.52
	2.0	2.0 ± 0.47	0.47	0.27	2.0 ± 1.2	1.21	0.7
	3.0	3.0 ± 0.51	0.5	0.29	3.0 ± 1.1	1.09	0.63

Each result is the average of three separate determinations

^a^ Within the day

^b^ Three consecutive days.

^c^ % Error = % RSD/ √n

The robustness of the method was evaluated by making minor deliberate changes in conditions of the experiment. The volume of buffer was varied by ± 0.2 mL and the pH by ± 0.2. There was no difference with those small variations which may take place throughout regular work. % Recoveries of the studied drugs in their commercial tablets were estimated to confirm the selectivity of the suggested method as presented in (Supplementary material Tables S2, S3). The results showed that common tablet excipients did not interfere with the analysis of both drugs, so the method is valuable for the estimation of EPL and SPR in their commercial tablets.

### Applications

#### Assay of EPL and SPR in their Pharmaceutical Tablets

The proposed method was successfully employed to estimate EPL and SPR in their commercial tablets. The results in (Supplementary material Tables S2 & S3) show excellent agreement with those obtained from the comparison methods [5, 12]. Variance F-test and Student’s t-test [33] were applied to assess the results obtained from the proposed method and comparison methods.

#### Assay of EPL and SPR in Spiked Human Plasma

**Fig. 5:**
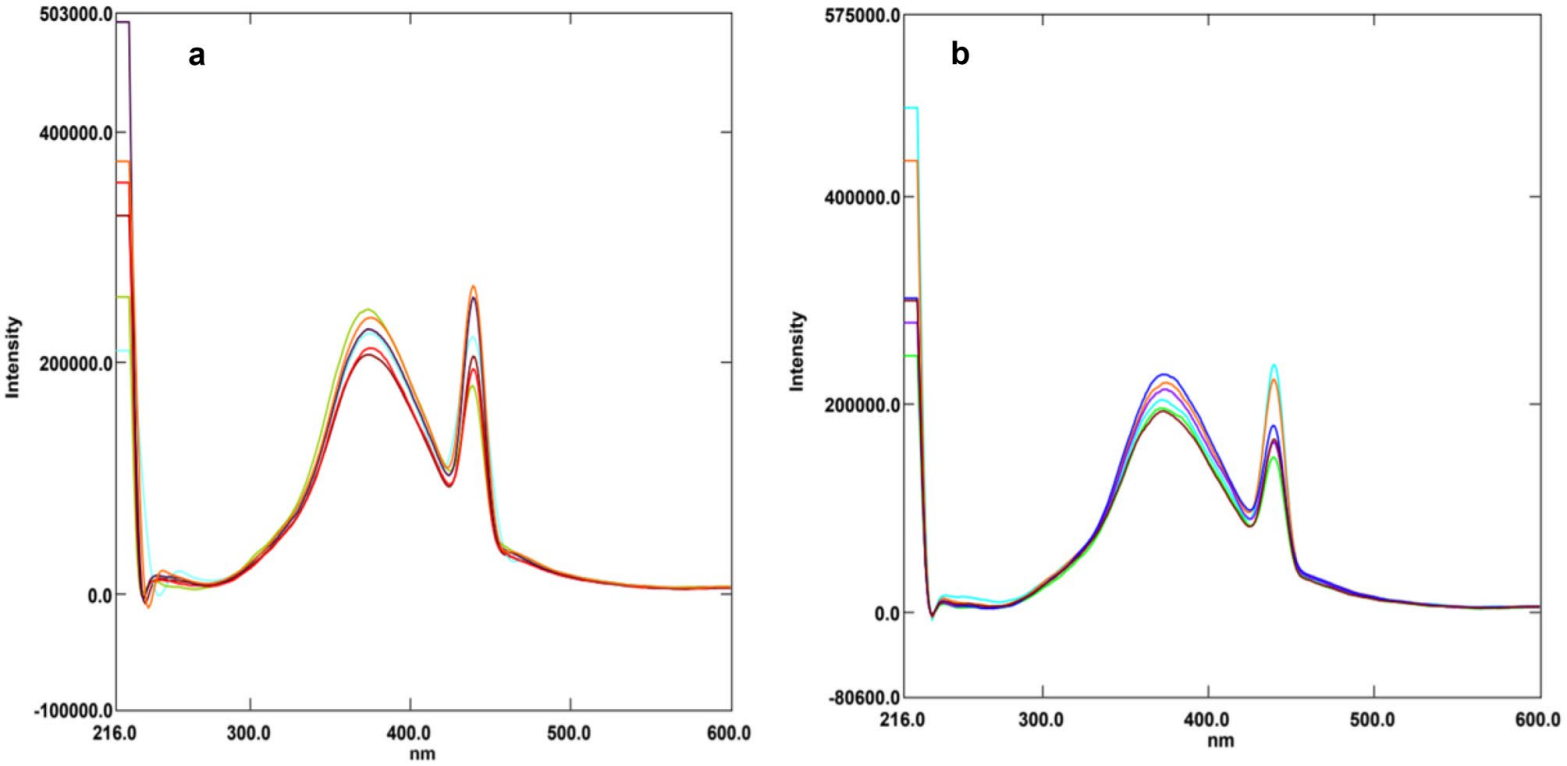
**a** Fluorescence emission spectra of the N-CQDs under optimum conditions upon spiking of various concentrations of EPL to human plasma. **b** Fluorescence emission spectra of the N-CQDs under optimum conditions upon spiking of various concentrations of SPR to human plasma

The proposed method was applied for the estimation of EPL and SPR in spiked human plasma samples to test the ability of the method to estimate both drugs in biological fluids. The peak plasma concentration after oral administration of 100 mg EPL is 1.72 μg/mL [34]. The preparation of plasma samples was carried out as mentioned before and then samples were analyzed. The percentage recovery values were 93.00 to 108.24% as shown in Table 4 and Fig. 5a. The suggested method can also be applied in case of acute toxicity of SPR in patients with impaired hepatic function as it may lead to hepatic coma [35]. The concentrations of SPR could be monitored in plasma after short time of acute toxicity. The percentage recovery values were 89.70 to 108.91% as illustrated in Table 4 and Fig. 5b.

**Table 4:** Application of the proposed spectrofluorimetric method for determination of EPL and SPR in spiked human plasma

Drug	Conc. added (µg/mL)	Conc. found (µg/mL)	%Recoveries
EPL	0.5	0.47	93.00
	0.8	0.87	108.24
	1.0	0.98	98.22
	1.5	1.48	98.38
	1.7	1.71	100.66
SPR	0.5	0.45	89.70
	1.0	0.93	92.80
	1.5	1.63	108.90
	2.0	2.15	107.73
	2.5	2.34	93.41

## Conclusion

The current study introduces a sensitive and rapid spectrofluorimetric method for the determination of EPL and SPR. The proposed method depends on utilizing N-CQDs as fluorescent probes for the quantitation of the studied drugs depending on the quenching effect of EPL and SPR on the fluorescence emission of N-CQDs without the need for any pre-derivatization steps. Cane sugar and urea were used as starting materials for the rapid microwave-assisted synthesis of the environmentally benign N-CQDs in about 10 min. The water-soluble N-CQDs with an average size of 7.7 nm showed strong fluorescence intensity and high stability. Furthermore, the prepared N-CQDs were applied as nano-sensors for the spectrofluorimetric determination of EPL and SPR in pharmaceutical tablets and spiked human plasma. The quantitation of EPL and SPR has been achieved over a wide range with LOQ of 0.383 µg/mL and 0.262 µg/mL and LOD of 0.126 µg/mL and 0.087 µg/mL. The proposed method has crucial characteristics such as low cost, simplicity, sensitivity and high selectivity.

### Supplementary Information

Below is the link to the electronic supplementary material.

Supplementary file1 (DOCX 328 KB)

## Data Availability

The datasets generated and/or analyzed during the current study are available from the corresponding author on reasonable request.

## Abbreviations

EPL: Eplerenone
SPR: Spironolactone
N-CQDs: Nitrogen doped carbon quantum dots
CQDs: Carbon quantum dots
BRB: Britton Robinson Buffer
IFE: Inner filter effect
