# Supplementary material for: Estimation of Two Diuretics Using Fluorescent Nitrogen Doped Carbon Quantum Dots: Application to Spiked Human Plasma and Tablets
Source: J Fluoresc. 2023 Mar 31;33(6):2209–18. doi: 10.1007/s10895-023-03217-z (PMC10640469; doi:10.1007/s10895-023-03217-z)
Supplement: Supplementary file 1 — Supplementary file1 (DOCX 328 KB) [file 10895_2023_3217_MOESM1_ESM.docx]

Supplementary Material

**Table S1**Assay results for the determination of the studied drugs in raw materials by the proposed and comparison methods

| **Compound** | **Proposed method** | | | **Comparison methods ^5,12^** |
| --- | --- | --- | --- | --- |
|  | **Concentration taken**  **(μg/mL)** | **Concentration found**  **(μg/mL)** | **% Found** | **% Found** |
| EPL | 0.5  1.0  2.0  3.0  4.0  5.0 | 0.49  1.01  1.99  3.04  3.92  5.04 | 98.23  101.45  99.59  101.41  98.05  100.77 | 100.58  99.42  100.19 |
| X̄ ± SD |  | | 99.92±1.53 | 100.06± 0.59 |
| *t-*test |  |  | 0.16 (2.36)* |  |
| *F*-test |  |  | 6.74 (19.29)* |  |
| SPR | 0.5  1.0  2.0  3.0  5.0  6.0 | 0.51  0.99  1.96  3.03  5.04  5.96 | 101.85  99.27  98.22  100.95  100.85  99.38 | 101.61  98.38  100.54 |
| X̄± SD |  | | 100.09±1.35 | 100.18±1.65 |
| *t-*test |  |  | 0.09 (2.36)* |  |
| *F*-test |  |  | 1.49(5.78)* |  |

**Table S2** Assay results for determination of EPL in its tablets by the proposed method

| **Preparation** | **Proposed method** | | | **Comparison methods^5^** |
| --- | --- | --- | --- | --- |
|  | **Amount taken**  **(μg/mL)** | **Amount found**  **(μg/mL)** | **% Recovery** |  |
| a-Tensopleron 25 mg tablets | 1.0 | 1.035 | 103.50 | 103.33 |
|  | 2.0 | 1.93 | 96.50 | 96.67 |
|  | 3.0 | 3.04 | 101.17 | 101.11 |
| ‾x |  | | 100.39 | 100.37 |
| ± SD |  |  | ±3.56 | ±3.39 |
| % RSD |  |  | 3.55 |  |
| % Error |  |  | 2.06 |  |
| t-test |  |  | 0.007(2.77)* |  |
| F-test |  |  | 1.1(19)* |  |
| b-Tensopleron 50  tablets | 1.0 | 0.98 | 102.28 | 98.25 |
|  | 2.0 | 2.03 | 97.18 | 101.75 |
|  | 3.0 | 2.98 | 100.94 | 99.42 |
| X̅ |  | | 99.82 | 100.13 |
| ± SD |  |  | ±1.61 | ±2.64 |
| % RSD |  |  | 1.61 |  |
| % Error |  |  | 0.93 |  |
| t-test |  |  | 0.17(2.77)* |  |
| F-test |  |  | 2.69(19)* |  |

*N.B. Each result was the average of three separate determinations.

*The figures between brackets were the tabulated *t* and *F* values at P = 0.05^33^.

a- Tablets contain 25 mg EPL.

b-Tablets contain 50 mg EPL.

**Table S3** Assay results for determination of SPR in its tablets by the proposed method

| **Preparation** | **Proposed method** | | | **Comparison methods^12^** |
| --- | --- | --- | --- | --- |
|  | **Amount taken**  **(μg/mL)** | **Amount found**  **(μg/mL)** | **% Recovery** |  |
| a- Spectone® 25 mg tablets | 1.0 | 0.998 | 100.55 | 100.55 |
|  | 2.0 | 2.004 | 99.45 | 99.45 |
|  | 3.0 | 2.997 | 100.18 | 100.18 |
| ‾x |  | | 99.97 | 100.06 |
| ± SD |  |  | ±0.24 | ±0.56 |
| % RSD |  |  | 0.24 |  |
| % Error |  |  | 0.14 |  |
| t-test |  |  | 0.25(2.77)* |  |
| F-test |  |  | 5.24(19)* |  |
| b-Spectone® 100  tablets | 1.0 | 1.013 | 100.58 | 98.25 |
|  | 2.0 | 1.974 | 99.42 | 101.75 |
|  | 3.0 | 3.013 | 100.19 | 99.42 |
| X̅ |  | | 100.15 | 100.06 |
| ± SD |  |  | ±1.33 | ±0.59 |
| % RSD |  |  | 1.33 |  |
| % Error |  |  | 0.77 |  |
| t-test |  |  | 0.098(2.77)* |  |
| F-test |  |  | 5.11(19)* |  |

*N.B. Each result was the average of three separate determinations.

*The figures between brackets were the tabulated *t* and *F* values at P = 0.05^33^.

a- Tablets contain 25 mg SPR.

b-Tablets contain 100 mg SPR.

**Figures**

**Fig S1** Chemical structure of eplerenone

**Fig S2** Chemical structure of spironolactone

**Fig S3** The UV–VIS absorption spectra of N-CQDs

**Fig S4** Fluorescence excitation and emission spectra of N-CQDs and N-CQDs after addition of 4 μg/mL of EPL

**Fig S5** Fluorescence excitation and emission spectra of N-CQDs and N-CQDs after addition of 1 μg/mL of SPR

**Fig S6** The UV–VIS absorption spectra of EPL and SPR

**Fig S7** Effect of pH on the fluorescence intensity quenching using 3 µg/mL of EPL and SPR

**Fig S8** Effect of volume of N-CQDs on the fluorescence intensity quenching

**Fig S9** Stern Volmer plot of F°/F against different drug concentrations at different Temperatures.

**Fig S10** Calibration curves of EPL and SPR

**
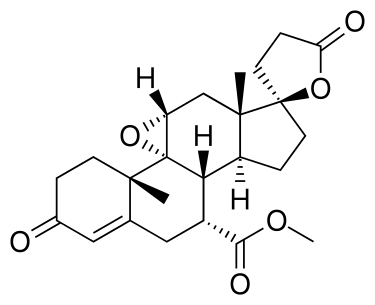
**

**Fig S1**

**
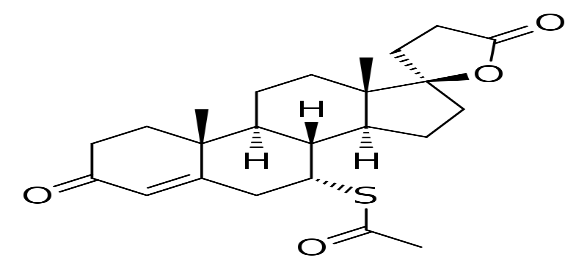
**

**Fig S2**

**
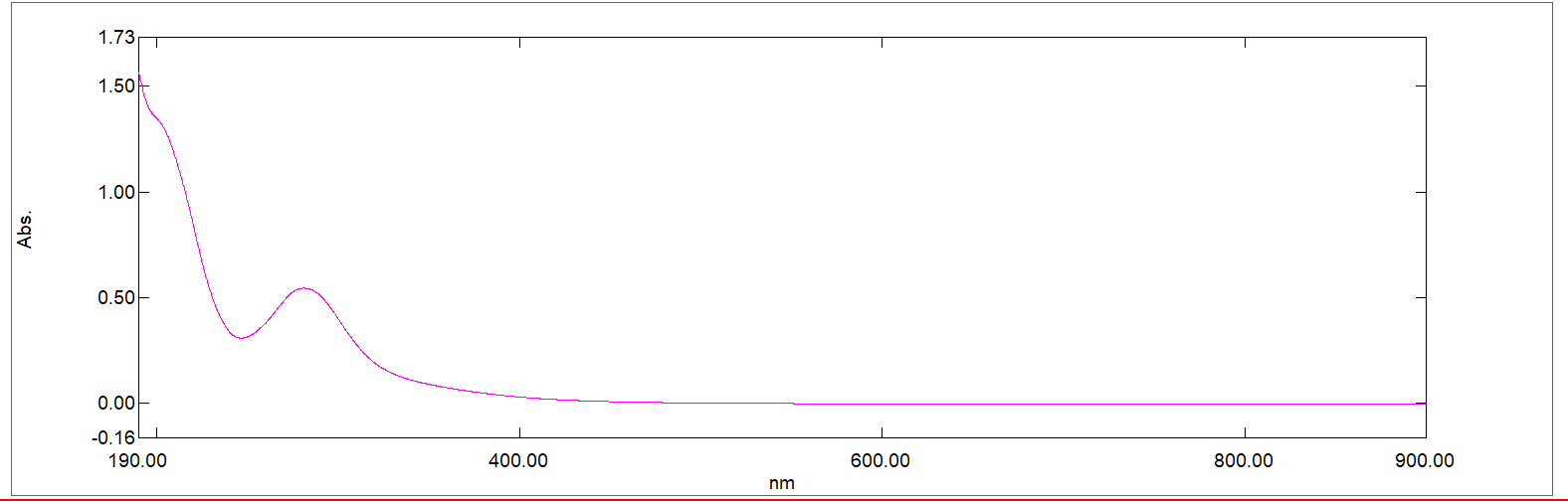
**

**Fig S3**


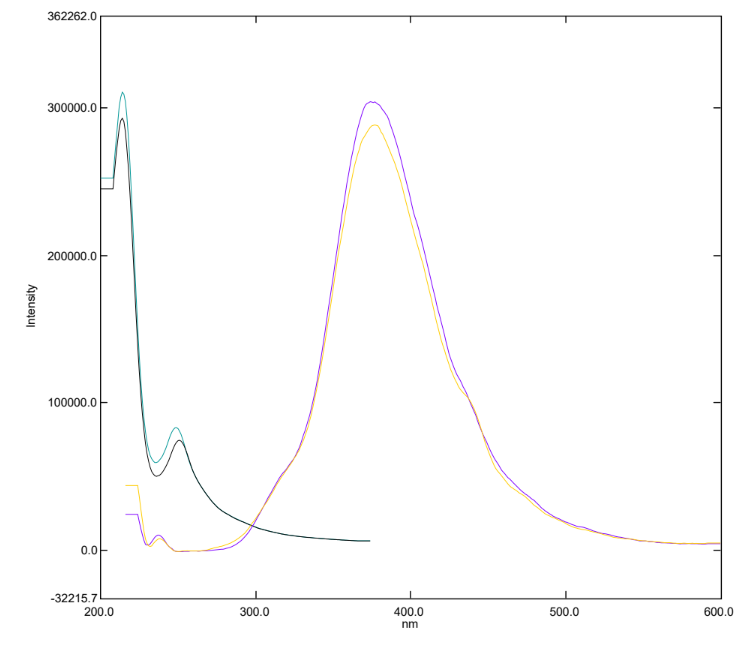


**Fig S4**

**
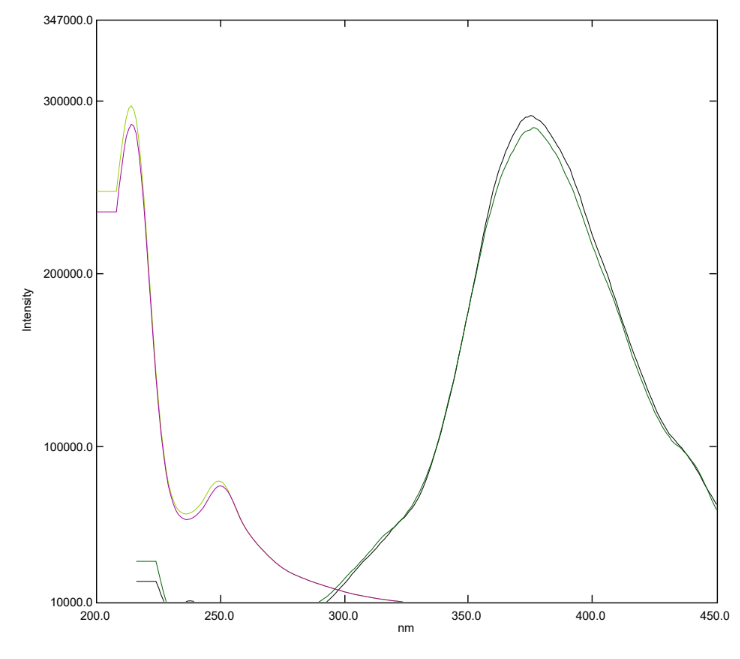
**

**Fig S5**

**
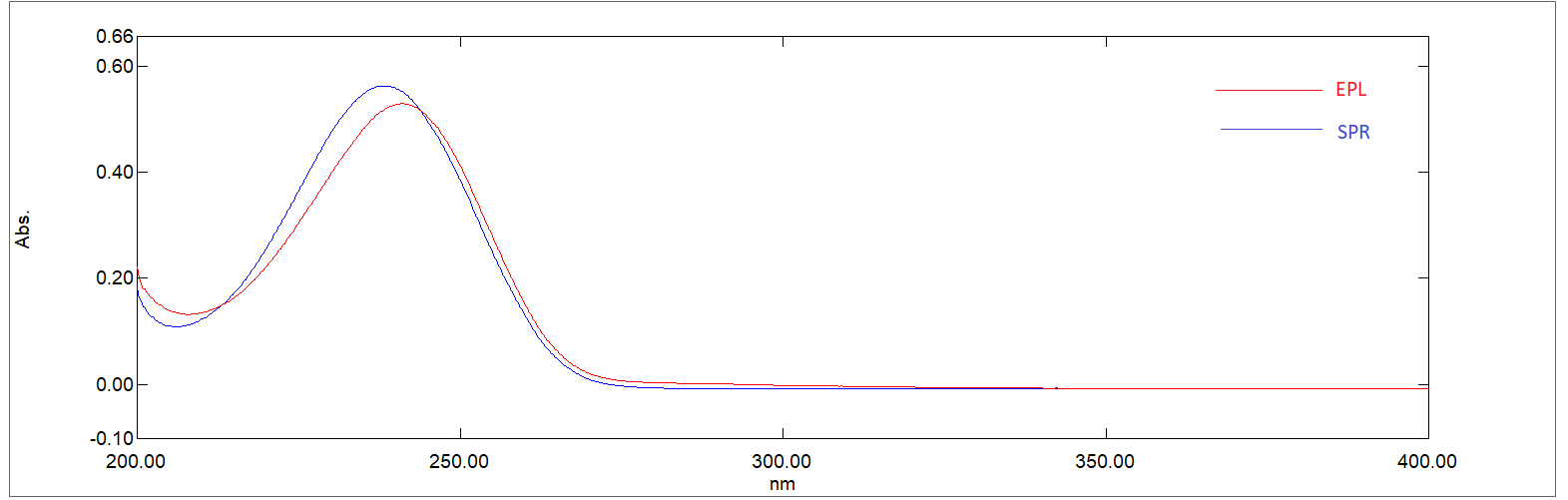
**

**Fig S6**

**
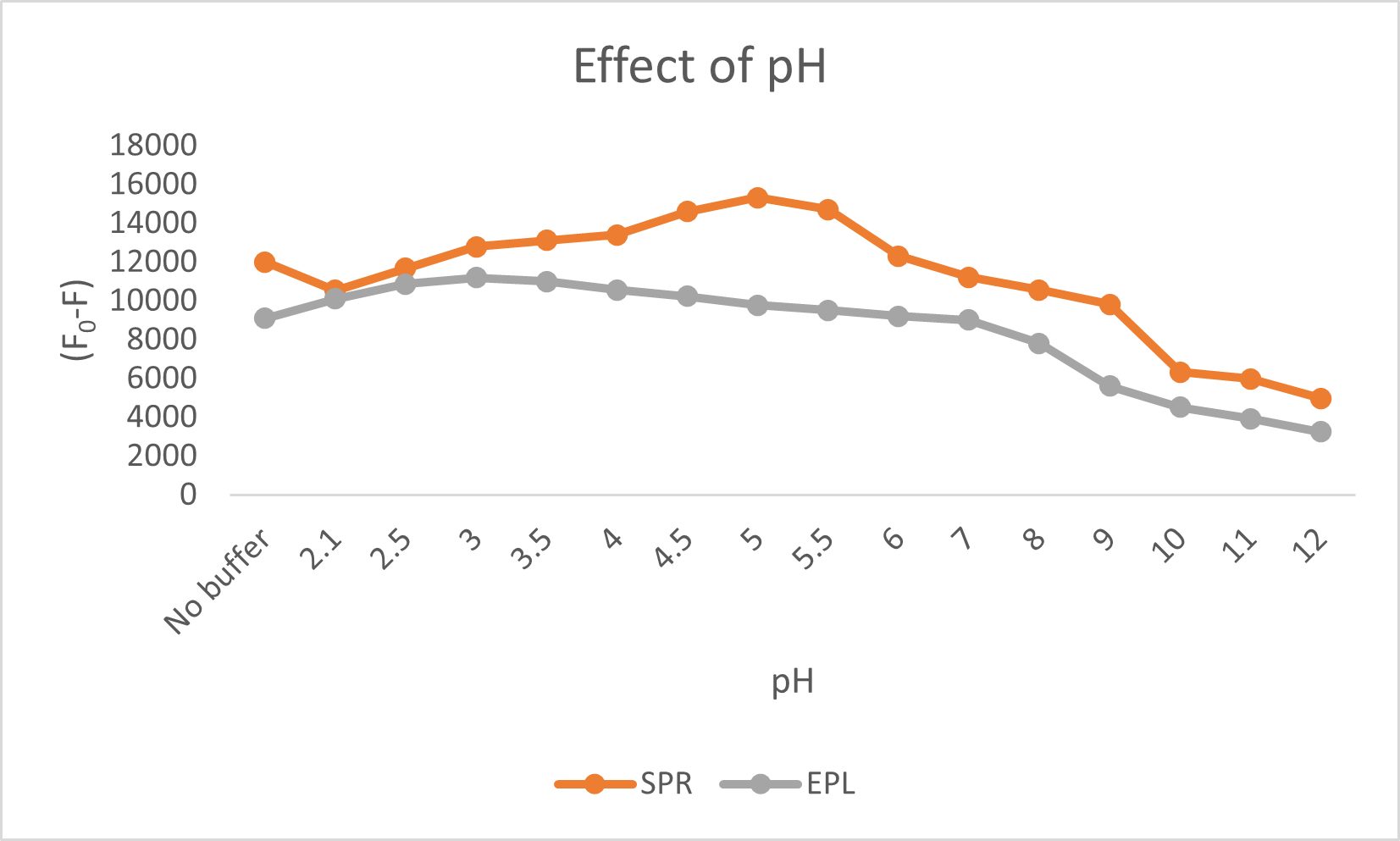
**

**Fig S7**

**
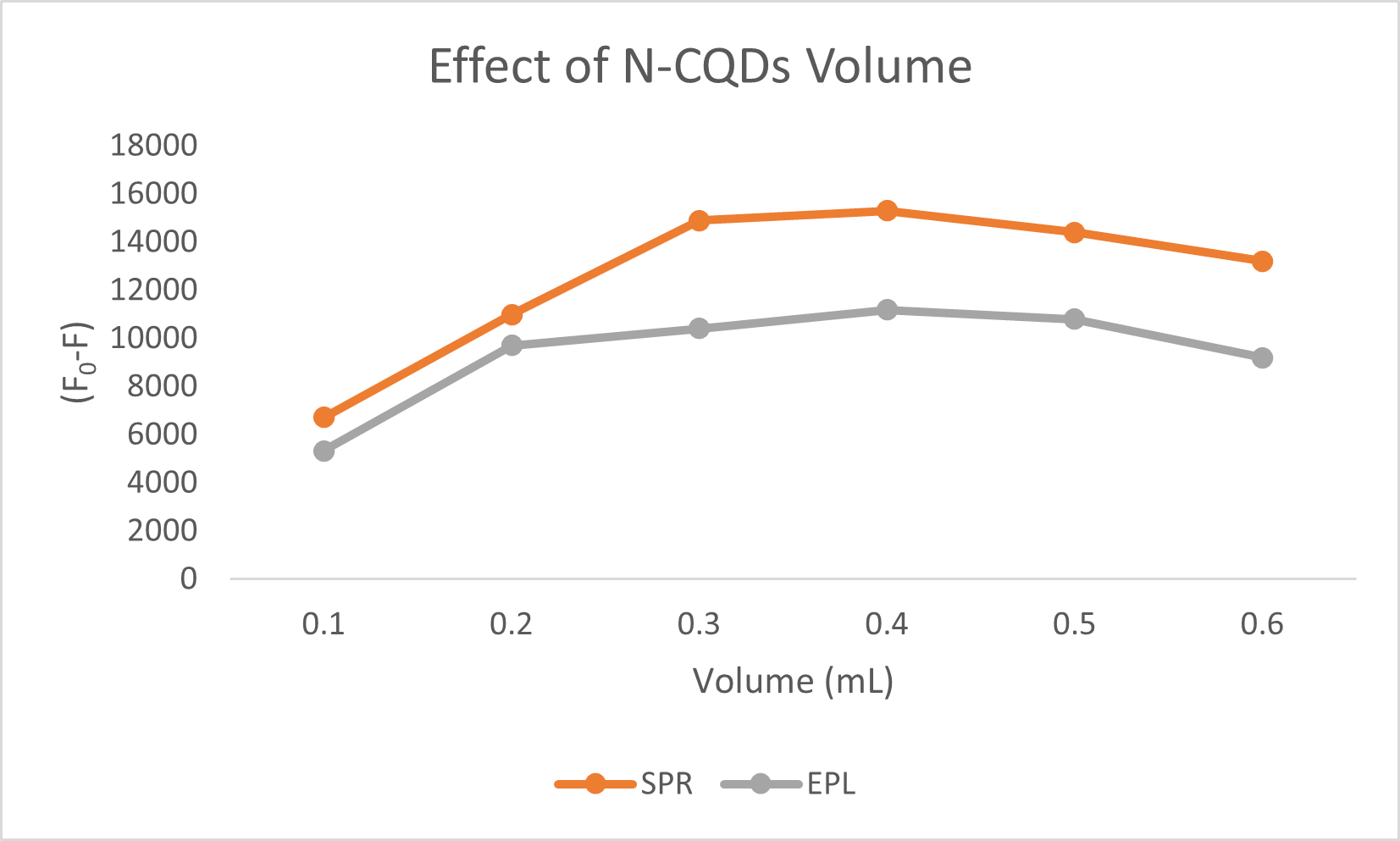
**

**Fig S8**

**
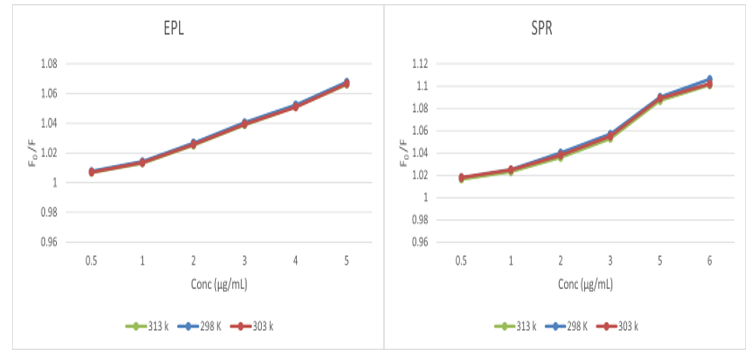
**

**Fig S9**

**
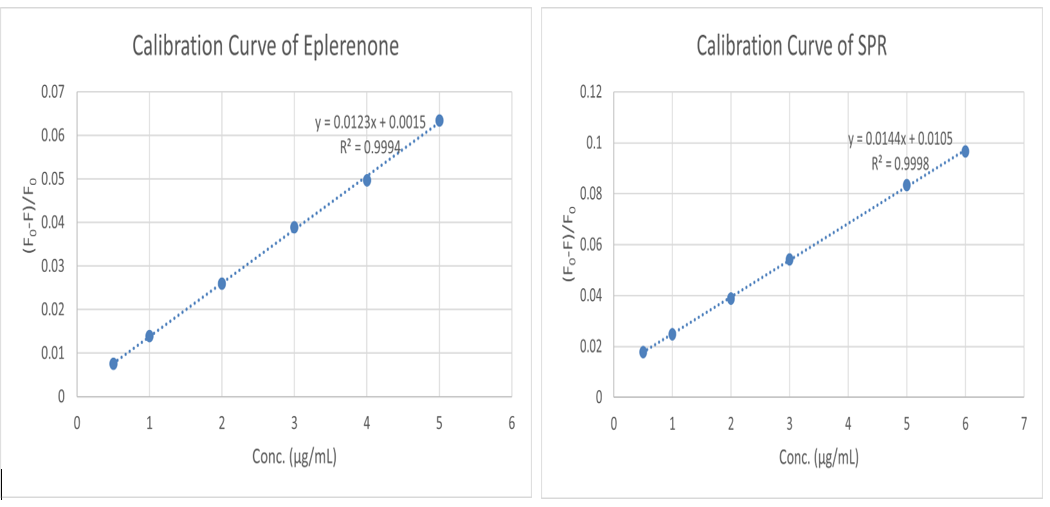
**

**Fig S10**
